# Genetic Variants Influence the Development of Diabetic Neuropathy

**DOI:** 10.3390/ijms25126429

**Published:** 2024-06-11

**Authors:** Noémi Hajdú, Ramóna Rácz, Dóra Zsuzsanna Tordai, Magdolna Békeffy, Orsolya Erzsébet Vági, Ildikó Istenes, Anna Erzsébet Körei, Peter Kempler, Zsuzsanna Putz

**Affiliations:** Department of Internal Medicine and Oncology, Semmelweis University, 1083 Budapest, Hungary; raczramona27@gmail.com (R.R.); tordai.dora@gmail.com (D.Z.T.); bekeffy.magdi95@gmail.com (M.B.); vagiorsi@gmail.com (O.E.V.); istildi78@gmail.com (I.I.); anna.korei@yahoo.com (A.E.K.); kempler.peter@med.semmelweis-univ.hu (P.K.); zsuzsannaputz@yahoo.com (Z.P.)

**Keywords:** diabetic neuropathy, risk factors, genetic variants

## Abstract

The exact mechanism by which diabetic neuropathy develops is still not fully known, despite our advances in medical knowledge. Progressing neuropathy may occur with a persistently favorable metabolic status in some patients with diabetes mellitus, while, in others, though seldom, a persistently unfavorable metabolic status is not associated with significant neuropathy. This might be significantly due to genetic differences. While recent years have brought compelling progress in the understanding of the pathogenetic background—in particular, accelerated progress is being made in understanding molecular biological mechanisms—some aspects are still not fully understood. A comparatively small amount of information is accessible on this matter; therefore, by summarizing the available data, in this review, we aim to provide a clearer picture of the current state of knowledge, identify gaps in the previous studies, and possibly suggest directions for future studies. This could help in developing more personalized approaches to the prevention and treatment of diabetic neuropathy, while also taking into account individual genetic profiles.

## 1. Introduction

The chronic complications of diabetes, from a didactic point of view, have been divided into microvascular and macrovascular complications for many years. Microvascular complications, including retinopathy, nephropathy, and neuropathy, are considered diabetes-specific. Macrovascular complications, including stroke, myocardial infarction, and peripheral arterial disease, are different manifestations of atherosclerosis. Among microvascular complications, neuropathy is of particular importance, which is a complication of diabetes with a poor prognosis. This condition develops over time in more than half of diabetic patients. Distal symmetric polyneuropathy (DSPN) is now the most important etiological factor of the diabetic foot. In developed industrialized countries, more than half of all non-traumatic lower-limb amputations are performed on people with diabetes, of which, in particular, those of neuropathic origin could be prevented by early detection and appropriate complex treatment [1,2,3,4].

Hospitalizations stemming from diabetic foot complications pose a significant and complex challenge to the healthcare system, which necessitates a multifaceted approach to address this pressing issue. For that reason, the early detection and effective management of sensory neuropathy emerge as elements of outmost importance in the holistic care of individuals with diabetes. Complying with effective metabolic management, practicing regular foot care, and conducting self-examinations of the feet have been acknowledged to increase patients’ quality of life, decrease treatment expenses by preventing amputations, and yield auspicious prognostic outcomes [5]. Moreover, pain and instability are the key factors contributing to reduced quality of life from neuropathy, whereas neuropathic damage is closely linked to an unfavorable outlook [5]. A meta-analysis by Vági et al. [6] of 31 cohort studies involving a total of 150,000 diabetic patients found an almost doubling of all-cause mortality among diabetes patients with distal symmetric polyneuropathy compared to those without DSPN. Presence of cardiovascular autonomic neuropathy (CAN) increases the mortality of patients by fivefold [7]. Vagi et al. [8] found that the presence of neuropathy signals an increased mortality risk, necessitating the strict control of conventional risk factors in affected individuals. They also rose the potential for improved survival through targeted treatments for diabetic neuropathy. They observed that individuals with type 1 diabetes mellitus combined with sensory neuropathy have higher relative mortality compared to patients with type 2 diabetes mellitus, but they need further research to confirm these findings. Overall, the study highlights the importance of recognizing CAN and DSPN as significant predictors of mortality and the necessity for comprehensive management strategies to reduce risks and enhance outcomes.

It is now clear that, in the prevention of microvascular complications such as neuropathy, near-normoglycemia, and close metabolic control, the management of other risk factors like hypertension, obesity, and hyperlipidemia is also very important. While strict glycemic control can lower the risk of complications, it alone is not enough to completely prevent them. This underscores the significance of genetic susceptibility in the development of the complications [9,10].

The research on biomarkers in diabetic neuropathy has intensified because of the difficulties in developing effective treatments. Biomarkers can have an important role in early detection, monitoring progression, and outlining treatment efficiency. Discovering dependable biomarkers could lead to more targeted therapies for this complication of diabetes mellitus.

## 2. Pathways Relevant to the Pathomechanism of Neuropathy

The exact mechanism by which diabetic neuropathy develops is still not fully understood, which has prompted researchers to explore the biomarkers of this complication. It has long been known that, in a group of people with diabetes, there is a relatively early onset of microvascular complications, while others do not develop this complication or, if they do, it occurs much later. It is likely that this difference is due to genetic factors. 

There are two main mechanisms involved in the development of neuropathy, functional and/or structural damage to the vasa nervorum, and the direct effect of hyperglycemia on neurons [11,12]. The pathogenetic significance of the metabolic pathway is under continuous investigation. Hyperglycemia leads to an increase in the amount of glucose entering nerve cells, which is compensated by the hexokinase capacity for a while. However, at the limit of maximum capacity, the alternative metabolic pathways, the polyol and hexosamine pathways, as well as mechanisms leading to protein kinase C (PKC) activation and enhanced end-glycation product (AGE) formation are amplified.

**Figure 1:**
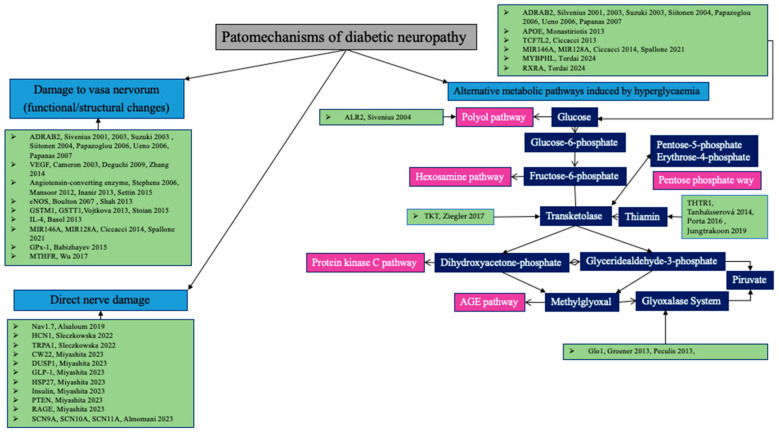
Potential genetic impacts on metabolic pathways contributing to the development of diabetic neuropathy, including the changes to vasa nervorum, the direct damage to the nerves, and the four alternative metabolic pathways induced by hyperglycemia. Hyperglycemia redirects metabolites from glycolysis into pathways like polyol, hexosamine, protein kinase C, and AGE. Transketolase, activated by thiamine, diverts metabolites into the pentose phosphate pathway. Methylglyoxal promotes AGE formation. The glyoxalase system metabolizes methylglyoxal. Loss of transketolase function weakens the defense against hyperglycemia-induced pathways in diabetic complications [13,14]. References on the Figure 1: [15,16,17,18,19,20,21,22,23,24,25,26,27,28,29,30,31,32,33,34,35,36,37,38,39,40,41,42,43,44,45,46,47,48,49,50].

The pentose phosphate pathway is another option to reduce the intracellular glucose load. Transketolase, of which a cofactor is vitamin B1, is the key enzyme in the latter pathway. Increasing enzyme activity stimulates the conversion of fructose-6-phosphate to pentose-5-phosphate, which may result in a reduction in the adverse effects of alternative metabolic pathways. Transketolase activity can be stimulated with thiamine or benfotiamine, thus exerting a protective effect against the four pathogenetic mechanisms of microvascular complications, the glucose-driven hexosamine pathway, the polyol pathway, protein kinase C, and the glycation end-product (AGE). A key enzyme in glycolysis is glycerol aldehyde-3-phosphate dehydrogenase. The activity of this enzyme decreases under hyperglycemia and oxidative stress, which results in the amplification of two metabolic pathways, the diacylglycerol protein kinase C activation and methylglyoxal-induced AGE production (Figure 1) [13,14].

Recent years have brought significant progress in the elucidation of the pathogenesis of neuropathy. Previous epidemiological studies have identified a number of risk factors such as age, female sex, physical work, lower education, and disadvantaged/poor living conditions [51,52]. Other risk factors include smoking, hypertension, obesity, hypercholesterolemia, and the duration of diabetes [53,54]. Based on the data currently available, it is evident that the potential genetic background plays a significant role in not only influencing the risk factors associated with diabetic neuropathy, but also in regulating the underlying mechanisms and pathways involved in this condition, as illustrated in detail in Table 1.

## 3. Genetic Variants Potentially Playing a Role in the Development of Diabetic Neuropathy

To date, not many human studies have investigated the genetic basis of neuropathy, and those analyzed are mostly single-nucleotide polymorphisms (SNPs). These studies have identified approximately 30 genes [60,61,62] that might play a role in the development of neuropathy (Table 1).

### 3.1. Angiotensin-Converting Enzyme (ACE)

The angiotensin-converting enzyme (ACE) is part of the renin–angiotensin system, which converts angiotensin I to angiotensin II, eliciting a powerful vasoconstrictor effect. ACE inhibitors have been used in diabetic neuropathy to repair microvascular damage, as some studies have shown a protective effect on neuronal dysfunction [63]. The *ACE* gene has an inversion/deletion (I/D) polymorphism, which can be classified into three types (II, ID, and DD). This polymorphism is determined by the deletion (D allele) or insertion (I allele) of the 287 bp Alu repeat in intron 16, and it determines ACE activity and the serum levels of the ACE. Several studies have demonstrated that there is a significant correlation between the homozygous DD genotype of the I/D polymorphism and the heightened risk of developing diabetic polyneuropathy [15,36,37,38].

### 3.2. Metylenetetrahydrofolate Reductase (MTHFR)

MTHFR catalyzes the methionine–homocysteine conversion. Different variants of the *MTHFR* gene reduce the activity of the enzyme. The C677T polymorphism in the *MTHFR* gene is the most common cause of elevated homocysteine levels [64]. Hyperhomocysteinemia has detrimental effects on the vascular endothelium and smooth muscle cells, leading to alterations in the arterial structure and function. Based on previous meta-analysis data, a clear association between the C677T polymorphism in the *MTHFR* gene and the risk of developing diabetic neuropathy has been demonstrated [16].

### 3.3. Glutathione S-Tansferase (GST)

Glutathione S-transferase (GST) protects against endogenous oxidative stress and exogenous potential toxins. GSTs can protect cells from oxidative damage, which is a feature of many pathological conditions, including neurodegenerative diseases and diabetic neuropathy. *GST-mu (GSTM1)* and GST-theta *(GSTT1)* and their polymorphisms are the most widely studied genes. The most common variant in the *GSTM1* and *GSTT1* genes is a homozygous deletion (null genotype), which is associated with reduced enzyme activity and cytogenetic damage [65]. A previous study has demonstrated that the combination of *GSTM1* and *GSTT1* genotypes significantly increases the risk of developing CAN in type 1 diabetic patients [17]; however, this has not been confirmed in type 2 diabetes [39].

### 3.4. Methylglyoxal

Methylglyoxal plasma levels have shown differing correlation with painful DPN or DPN in type 2 diabetes in cross-sectional studies [66,67,68]. Nevertheless, in the ADDITION-Denmark cohort, methylglyoxal plasma levels were found to independently predict the incidence of DPN, with a hazard ratio of 1.46 (95% CI 1.12–1.89) [69]. Moreover, methylglyoxal modifies the nociceptor-specific Na channel (Nav1.8), enhancing the excitability of sensory neurons, which leads to hyperalgesia [66]. 

In the same way, methylglyoxal exhibited the ability to stimulate the transient receptor potential cation channel subfamily A, member 1 (TRPA1) [70,71]. TRPA1 is a receptor channel found in sensory neurons and is associated with inflammatory and neuropathic pain. The amendment caused by methylglyoxal in sodium and transient receptor potential channels could potentially lead to the development of neuropathic pain.

### 3.5. Glioxalase (GLO)

In association with DPN, the genetic variation within the glyoxalase system has been studied. Specifically, one of the identified *SNPs* in the *GLO1* gene (*GLO1*), which involves an adenosine/cytosine switch at nucleotide position 332 (rs2736654 or rs4746), has garnered significant interest. This particular SNP results in the substitution of alanine with glutamic acid at protein residue 111 (changing from C332-111Ala to A332-111Glu), potentially impacting the morphology of the glyoxalase binding site. Glo1-Ala/Ala (C332C), Glo1-Ala/Glu (C332A), and Glo1-Glu/Glu (A332A) are the three phenotypes of the Glo-1 enzyme that have been identified. The decrease in Glo-1 activity was observed in a culture of immortalized lymphoblastoid cells, which were homozygous for the A allele, accompanied by elevated levels of intracellular methylglyoxal and the receptor for AGEs [72].

In a study by Groener et al. [18], the SNP rs2736654 was examined in 209 patients with type 1 diabetes and 524 patients with type 2 diabetes to investigate its connection to the complications of diabetes mellitus. The study revealed a significantly higher prevalence of the A332A genotype in type 1 diabetes in comparison to type 2 diabetes (35.9% vs. 27.3%; *p* = 0.03). However, in participants with type 1 diabetes mellitus, no association was found between any genotype and diabetic neuropathy, nephropathy, or retinopathy. In turn, the C332C genotype correlated with DPN in patients with type 2 diabetes; the 53.7% of carriers of the C332C genotype had diabetic neuropathy in comparison to the 44% of carriers of the A332A and C332A genotypes (*p* = 0.03; odds ratio = 1.49; 95% confidence interval, 1.04–2.11). Although this association did not hold after correction for multiple comparisons, multiple logistic regression analysis demonstrated an independent correlation of the C332C genotype with diabetic neuropathy in patients with type 2 diabetes mellitus (*p* = 0.018) rather than with diabetic nephropathy or retinopathy. This study represents the first comprehensive, cross-sectional investigation indicating a potential link between the *GLO1* polymorphism and diabetic neuropathy in patients with type 2 diabetes mellitus, displaying the involvement of methylglyoxal in the pathogenesis of diabetic neuropathy, particularly in type 2 diabetes mellitus. The A332A genotype, rather than the C332C genotype, was formerly linked to decreased glyoxalase activity [72], which did not align with the hypothesis of the reduced detoxification capacity being pathogenetically significant. Groener et al. proposed several potential reasons for this inconsistency, including the use of inadequate surrogate markers to measure Glo-1 activity in the earlier research, the potential influence of unknown genetic factors linked to *GLO1* polymorphisms that could alter the prospect of diabetic neuropathy, and the complex nature of methylglyoxal detoxification mechanisms.

The glyoxalase system also has a prominent role in the pathogenesis of diabetic complications. This system is composed of glioxalase 1 (Glo-1), glioxalase 2 (Glo-2), and glutathione, and it acts as a defense against AGE formation. In particular, genetic variants in the *GLO1* gene may cause changes in the structure of the glyoxalase binding site [28]. The study by Peculis et al. [28] was the first to document the association between rs1130534 and rs1049346 SNPs and reduced Glo-1 enzyme activity. On the other hand, the increased frequency of the CC genotype of the *GLO1* gene (rs2736654 or rs4746) has been reported in patients with diabetic neuropathy [18].

In a different study involving 326 participants (101 subjects with type 1 diabetes, 100 subjects with type 2 diabetes, and 125 healthy subjects), the study examined the correlation between Glo-1 activity in whole-blood lysates and the same SNP in *GLO1* rs2736654, along with two other frequent SNPs, rs1130534 (G124G) and rs1049346 (5′-UTR). The study found that blood Glo-1 activity was reduced in individuals with the rs1130534 AT and TT genotypes, as well as the rs1049346 TT and CT genotypes. Each T allele of rs1130534 and rs1049346 was associated with a reduction of 3.1 U/g Hb and 2.8 U/g Hb in blood Glo-1 activity, correspondingly. These findings remained statistically significant even after adjusting for multiple testing. The SNP rs2736654 did not show a significant correlation with Glo-1 activity as predicted by whole-blood lysates [28]. However, individuals with the C332C genotype tended to exhibit lower Glo-1 activity levels. These findings conflicted with the results of a study by Barua et al. [72], which utilized a different experimental approach and population. These results may provide some back-up for the link between the C332C genotype and diabetic peripheral neuropathy as examined by Groener et al. [18]. The research conducted by Peculis et al. [28] was the first to reveal a connection between SNPs rs1130534 and rs1049346 and diminished Glo-1 enzyme activity. When exploring the influence of the glyoxalase/methylglyoxal system and its genetic variations on the pathogenic mechanisms of DPN, it is essential to consider not only the methodological challenges in measuring methylglyoxal but also the existence of alternative detoxification systems apart from glyoxalase and various compensatory pathways [73]. In conclusion, determining a link between genetic variations in the *GLO1* gene and alterations in glyoxalase activity and methylglyoxal levels presents a challenging objective.

### 3.6. Apoliporotein E (APOE)

The three isoforms of the apolipoprotein E (*APOE*) gene play an important role in the cholesterol and triglyceride metabolism. The presence of the Ɛ4 allele of the *APOE* gene appears to increase the risk of severe diabetic neuropathy [19]. The transcription factor 7-like 2 *TCF7L2* gene affects the lipid metabolism and glucose homeostasis. Analysis of three polymorphisms of the *TCF7L2* gene (rs7903146, rs7901695, and rs12255372) demonstrated a strong correlation between rs7903146 and CAN [20].

### 3.7. Vascular Endothelial Growth Factor (VEGF)

Human vascular endothelial growth factor (VEGF) facilitates the proliferation of vascular endothelial cells. In recent years, VEGF levels have been reported to increase in the presence of diabetic neuropathy [21,40]. The presence of the 936C/T mutation of the *VEGF* gene further stimulates the risk of developing diabetic neuropathy, while the presence of the T allele decreases it [41].

### 3.8. Interleukin-4 (IL-4)

Intrleukin-4 (IL-4) is an important cytokine that impacts immune cell chemotaxis and anti-inflammation. The *IL-4* gene VNTR (variable number of tandem repeat) polymorphism plays an important role in the occurrence of diabetic neuropathy [22]. GPX1 is an antioxidant enzyme. A polymorphism in the gene (rs1050450, C > T) results in an amino acid change from proline to leucine in codon 198, reducing the enzyme activity. The rs1050450 T allele is also a genetic risk factor for diabetic neuropathy [55].

### 3.9. Endothelial Nitric Oxide Synthase (eNOS)

Endothelial dysfunction has an impact on the development of microvascular complications. Endothelial nitric oxide synthase (*eNOS*) is responsible for the synthesis of nitric oxide. The *eNOS* gene polymorphisms that lead to reduced eNOS expression have been suggested to be associated with the development of diabetic neuropathy [42]. The two most studied SNPs, rs2070744 (786 T/C) and rs1799983 (894 G/T), are considered to be genetic predisposing factors for the development of neuropathy [23].

### 3.10. Adrenoceptor Alpha 2B (ADRA2B)

A common non-synonymous mutation in the adrenoceptor Alpha 2B (*ADRA2B*) gene (12Glu9) encodes a receptor protein that causes the I/D polymorphism of three consecutive glutamates in the 301–303 positions. The mutation has been associated with metabolic and vascular effects, including obesity, reduced insulin secretion, and the development of diabetes [24,43,44,45,46,47,74]. That this I/D polymorphism in the nervous system is associated with autonomic dysfunction and increased sympathetic nervous system activity supports a potential role for this polymorphism in the development of diabetic neuropathy. Exploring the potential association between the *ADRA2B* gene I/D polymorphism and diabetic neuropathy, a higher prevalence of the D allele has been reported in patients with neuropathy, suggesting that the presence of the D allele plays a role in the severity of this condition.

### 3.11. MicroRNA (MIR146A, MIR128A, MIR499A)

Investigating the role of polymorphisms in microRNA (MIR) regions in the development of diabetic neuropathy, rs2910164 (G > C) in *MIR146A* and rs11888095 (C > T) in *MIR128A* have been found to be in correlation with the risk of developing disease [25]. The rs2910164 variant in *MIR146A* is accompanied with a lower risk of diabetic neuropathy, while the presence of rs11888095 in *MIR128A* is associated with a higher one. Spallone et al. [48] also found a correlation between the rs3746444 SNP in the *MIR499A* gene (GG genotype) and DPN. This phenotype was associated with the decreased copy number of mitochondrial DNA; therefore, damage to the mitochondrial biogenesis could be shown, which in turn lowered the defense against oxidative stress and the hyperglycemic load.

### 3.12. Thiamine Transporters (THTR1/THTR2)

It is possible that the thiamine pathway is relevant to the development of diabetes complications [75]. Intracellular transport of thiamine is regulated by two thiamine transporters (THTRs), THTR1 and THTR2. Genetic tests have identified mutations in the gene solute carriers (SLCs), *SLC19A2* and *SLC19A3,* encoding THTR1 and THTR2, respectively, which may be responsible for the development of neurological conditions. THTR1 defects may lead to mitochondrial dysfunction, and thus lowered defense against oxidative stress and cell cycle arrest [26]. 

Both a deficiency in thiamine, caused by the misconduction of thiamine in the kidneys, and a reduced ability to effectively regulate thiamine transporters contribute to the worsening of the metabolic effects of high blood sugar levels and the development of diabetic complications. Studies have pinpointed mutations in the *SLC19A2* and *SLC19A3* genes that control the production of THTR1 and THTR2 proteins. A loss-of-function mutation in the *SCL192* gene leads to a severe genetic disorder known as thiamine-responsive megaloblastic anemia (TRMA) or a form of diabetes similar to maturity-onset diabetes of the young (MODY). On the other hand, variations in the *SCL19A3* gene are linked to well-known neurological disorders such as biotin-responsive basal ganglia disease (BBGD) and biotin–thiamine-responsive basal ganglia disease (BTBGD). These conditions manifest in the following three main forms: classical childhood BBGD, early-infantile Leigh-like syndrome/atypical infantile spasms, and adult Wernicke’s-like encephalopathy. These genetic conditions underscore the importance of adequate thiamine levels for proper neuronal and neuromuscular function, in addition to its essential role in various metabolic processes.

In a study conducted by Porta et al. [49], genetic variations in genes encoding thiamine transporters and their associated transcription factors SP1/2 were examined in regard to progressed retinopathy, nephropathy, or a combination of both in patients with type 1 diabetes from the Finnish Diabetic Nephropathy (FinnDiane) cohort. The findings were further validated in cohorts from the DCCT/EDIC and Wisconsin Epidemiologic Study of Diabetic Retinopathy (WESDR). Out of the 134 single-nucleotide polymorphisms (SNPs) analyzed, 2 SNPs within the *SCL19A3* locus, specifically rs12694743 and rs6713116, were identified as being significantly associated with a protective effect against severe retinopathy (*p* = 3.8 × 10^6^; odds ratio 0.51; 95% CI, 0.38–0.68), and the combined occurrence of severe retinopathy and end-stage renal disease (ESRD) (*p* = 7.5 × 10^−8^; odds ratio 0.31; 95% CI, 0.20–0.47). Notably, the association with the combined phenotype reached genome-wide significance in a meta-analysis that integrated data from the WESDR cohort (*p* = 2.3 × 10^−8^; odds ratio 0.28; 95% CI, 0.18–0.44). These SNP associations with diabetic retinopathy and nephropathy persisted even after adjusting for covariates, suggesting that these SNPs may represent a new independent risk factor for these complications.

### 3.13. Transketolase (TKT)

Genetic variation in the transketolase gene was investigated in a study involving 240 subjects with diabetes (including type 1, latent autoimmune diabetes in adults (LADA), and type 2 diabetes), with or without diabetic nephropathy, including end-stage renal disease (ESRD) in some cases [76]. The research revealed a notable thiamine deficiency in diabetic patients with kidney disease, but did not establish a significant association between transketolase gene polymorphisms and both transketolase erythrocyte activity and the presence of diabetic nephropathy after applying Bonferroni correction for multiple comparisons. In a subsequent 38-month prospective study by the same authors involving 314 patients with type 2 diabetes and diabetic nephropathy (including 42 with ESRD), the impact of 19 SNPs in six genes related to enzymes metabolizing glycolytic intermediates (such as transketolase, transaldolase, transketolase like-1, fructosamine 3-kinase, glyoxalase 1, and glucose-6-phosphate dehydrogenase) was assessed. The results indicated that the transketolase SNP rs11130362 and the fructosamine 3-kinase SNP rs1056534 together had a significant effect on nephropathy progression (*p* = 0.00645). Additionally, the transketolase SNP rs3736156 on its own (*p* = 0.00442) and in combination with the two aforementioned SNPs had a notable impact on the occurrence of major cardiovascular events (*p* = 0.01014) [50].

Ziegler et al. [27] investigated the role of transketolase (TKT) genetic variability in the development of neuropathy. Transketolase is a rate-limiting enzyme of pathways proposed to confer hypothetical protection against hyperglycemia, with neuropathic symptoms and reduced thermal sensation in recently diagnosed diabetes.

The study included 165 type 1 and 373 newly identified type 2 diabetic patients. Altogether, 13 SNPs were selected in the *TKT* gene, and they found several associations between SNPs and peripheral nerve function [27]. However, most of these correlations lost significance after Bonferroni correlation, except for the correlations between the rs7648309 SNP and the symptom score, as well as the rs63355988 SNP and the warmth perception. The study was the first to demonstrate a link between diabetic neuropathy and some TKT SNPs. The results suggest that TKT may have a protective action in the prevention of diabetic neuropathy. Based on these studies, pharmacogenomics considering transketolase SNPs could be useful to optimize such treatments. In the future, the parameters of the thiamine metabolism should be measured to explore a possible genotype–phenotype interaction between the latter and transketolase SNPs in relation to diabetic neuropathy. As well, as the cross-sectional study design did not allow for the determination of the predictive value of transketolase SNPs on the development and progression of diabetic neuropathy, which has to be verified in long-term prospective studies.

### 3.14. Ion Channels

A case study of a male patient with painful diabetic neuropathy, aspartic acid–aspartic acid mutation (D109N) in the voltage-dependent Na channel beta-2 subunit of Nav1.7, was reported, resulting in the hyperexcitability of posterior ganglion neurons [29]. Neuropathic pain causes poorer quality of life in people with diabetes [77]. Preceding studies had found several risk factors for neuropathic pain like female sex, smoking, age, weight, and longer diabetes duration [78,79,80]. Previously, genetic variants of the voltage-gated sodium ion channels (VGSCs) have been identified with the use of next-generation sequencing (NGS), which could be connected to neuropathic pain [79,81]. These genes have an important part in the generation and spreading of the action potential in nociceptors and on the nerve fibers [29,81,82,83,84,85].

Therefore, Sleczkowska et al. [30] inquired into the role of ion channels in painful diabetic neuropathy. They analyzed the voltage-gated potassium (Kv), transient receptor potential (TRP), anoctamin *(ANO)*, and hyperpolarization-activated and cyclic nucleotide-gated channel *(HCN)* ion channel genes that are expressed in peripheral nerves. They used single-molecule inversion probes and next-generation sequencing (NGS). They found that mis-sense heterozygous variants in the *ANO3* and *HCN1* genes and TRPA1 loss-of-function are linked to increased pain sensitivity. They also demonstrated that variations in the *TRPV1* and *TRPV4* genes that lead to loss-of-function might be present in painless diabetic neuropathy. 

In another study, the role of the potential pathogenic single-copy gene (SCG) genetic variants in painful and painless diabetic neuropathy, as well as in painful and painless idiopathic neuropathy, was explored [31]. They profiled 1125 patients (237 painful and 309 painless diabetic neuropathies, 547 painful small-fiber neuropathies, and 32 painless single-fiber neuropathies) with a single-molecule inversion probe and NGS. They discovered an association between gain-of-function mutations in the sodium channel (SCN) *SCN9A*, *SCN10A*, and *SCN11A* genes and neuron hyperexcitability, and thus with pain.

### 3.15. Glia Cell Line-Derived Neurotrophic Factor Family Receptor Alpha-2 (GFRA2)

A multicenter study [56] that looked at about a million SNPs in the whole genome found a single region (chromosome 8 p21.3) that showed an association with neuropathy. In this study, however, the presence of neuropathy was based on whether the patient had taken a drug for neuropathy and/or the monofilament test was abnormal. In the genomic locus found, nine SNPs showed significant correlations. These SNPs were intergenic SNPs adjacent to the Glia cell line-derived neurotrophic factor (GDNF) family receptor alpha-2 (GFRA2) and the neurturin receptor gene. The GFRA2 protein is a glycosylphosphatidylinositol-coupled cell-surface receptor which is a member of the GDNF receptor family. GDNF is a factor that plays an essential role in the differentiation and survival of neurons. The GFRA2 receptor binds to this family of proteins, and proper receptor function is required for proper action. The receptor activates the RET tyrosine kinase receptor pathway [86]. Based on this, it is possible that genetic polymorphisms in the *GFRA2* gene may determine the susceptibility to diabetic neuropathy.

### 3.16. Aldose Reductase (ALR)

The aldose reductase (*ALR*) gene expression could be produced by methylglyoxal (MGO), AGEs, and oxidative stress caused by the hyperglycemic state. This gene has a complex role the complications of diabetes mellitus. Sivenius et al. [32] found that a 106C/T polymorphism in the promoter region of the *ALR2* gene is connected to the decrease nerve conduction velocities of the motor peroneal nerve in patients with type 2 diabetes mellitus, while the 106C/C genotype was associated with lower amplitudes of the sensory nerves. 

Other authors found a polymorphism located at 5’ in the upstream regulatory region of ALR2, the 50-(CA)n microsatellite polymorphism that has more than 10 alleles, which also collated with diabetic neuropathy. There are two main alleles, the Z − 2 and Z + 2, where Z equates to 24CA repeats. In the study, they found that the Z + 2 allele seemed to protect against diabetic neuropathy, while the Z − 2 allele was associated with higher susceptibility to complications in both type 1 and 2 diabetes mellitus [57].

### 3.17. Glutathione Peroxidase 1 (GPx-1)

In the gene of glutathione peroxidase 1 (GPx-1), a polymorphism 599C/T (rs1050450) has been shown to be associated with DN. They found the same correlation in the 262C/T in the gene catalase (CAT) [33]. In a genome-wide association study, Meng et al. found an association between neuropathic pain and chromosomal loci 1p35.1 and 8p21.3 [58].

### 3.18. Results of the First Whole-Exome Sequencing Study

In our recent study [35], 24 patients with long-term type 2 diabetes with neuropathy and 24 without underwent a detailed neurological assessment and whole-exome sequencing. We could successfully identify genetic variants that might alter the risk of developing diabetic neuropathy. The rs604349 is an intronic SNP in *MYBPHL* (myosin-binding protein H-like) gene that seems to aggravate the risk for neuropathy. This gene has been linked to circulating progranulin. The rs2032930/rs2032931 are intronic SNPs found in the *RMI2* (recQ-mediated genome instability protein 2) gene, and appeared to increase the risk of developing neuropathy. In our study, rs917778 and rs2234753 were accompanied with a reduced risk for diabetic neuropathy. The rs917778 is also an intronic SNP in the *MVB12B* (multivesicular body subunit 12B) gene. Another genetic variant with a reduced risk for diabetic neuropathy is rs2234753. It is also an intronic SNP in the *RXRA* (retinoic acid X receptor alpha) gene. In summary, all five SNPs that have been demonstrated to interfere with the risk of diabetic neuropathy in our study can be found in an intronic region of the genes, i.e., they do not become transcribed. Nevertheless, these variants might be part of higher-level regulating systems that indirectly influence pathophysiological processes that may affect the development of neuropathy. Once our data are further corroborated, we might be able to establish new strategies for early preventive intervention and identify targets for new drug developments in the future.

### 3.19. Others

In a review conducted by Zhao et al., a systematic analysis was performed on a total of 1256 articles. From these, 106 publications detailing 136 polymorphisms of 76 genes were identified. Although the study had some limitations, it revealed associations between ACE I/D, MTHFR 128A/C, GPx-1 rs1050450, and CAT-262C/T and the susceptibility to diabetic neuropathy [87]. In their review article, Jankovic et al. detailed the key gene polymorphisms associated with diabetic neuropathy that were previously listed [59]. Furthermore, this review study investigated epigenomic mechanisms such as DNA methylation. Hyperglycemia induced the changing DNA methylation status in white blood cells, which can be used as a potential biomarker for PDN. Moreover, the NINJ2 (ninjurin 2) protein helps Schwann cells to regenerate after an injury. The decreased expression of NINJ2 was found after an increased methylation, which may contribute to neuropathy development. Some pathways—nervous system development and/or axon guidance (netrin-4 (*NTN4*) and dihydropyrimidinase-like 2 (*DPYSL2*) genes, the glycerophospholipid metabolism (phospholipase and phosphatidylserine decarboxylase), and MAPK signaling—have differences in the progression of PDN by DNA methylation profiles with PDN progression. Epigenomic mechanisms also include microRNAs that are noncoding RNAs of less than 200 nucleotides in length. The following microRNAs have been associated with the development of diabetic neuropathy: miR9, miR199a3p, miR25, miR146, and miR190a5p. Moreover, long noncoding RNAs (more than 200 nucleotides in length) have been associated with neuropathy through the MAPK signaling pathway (CCNT2-AS1, RP1-249H1.2 CTD-3239E11.2, RP11-51B23.3, STAM-AS1, and LINC00629). Last, but not least, post-translational histone modification may play a role in neuropathic pain and peripheral nerve injury-induced neuropathic hypersensitivity.

Miyashita et al. discusses the importance of activating neurotrophic effects in insulin/PI3K/pAkt signaling to enhance the regenerative capacity of diabetic neurons in response to ongoing degeneration [34]. The study explains the mechanisms of insulin resistance in sensory neurons in type 1 and type 2 diabetes, as well as the potential benefits of intrathecal injections of insulin and the neurotrophic effect of glucagon-like peptide-1 (GLP-1). Moreover, the research explores the role of molecules like PTEN and Heat Shock Protein 27 (HSP 27) in regulating neuronal growth and protection, as well as the impact of chronic hyperglycemia and advanced glycation end-products (AGEs) on DPN. Miyashita et al. discussed the involvement of the receptors for AGEs (RAGE) in diabetic neuropathy and the potential dual roles of the AGE-RAGE signaling pathway. The research also referred to changes in global gene expression within the dorsal root ganglion (DRG) sensory neurons in diabetes, introducing differentially expressed mRNAs and their potential therapeutic implications, such as CWC22 and DUSP1, in improving neuropathic features in diabetes. 

## 4. Conclusions

Diabetic polyneuropathy is a miscellaneous complication of diabetes mellitus that can have a profound impact on the morbidity and mortality of individuals living with diabetes. The development and progression of diabetic neuropathy display significant variability among patients, suggesting that, beyond just metabolic factors, genetic predisposition may also play a crucial role in its pathogenesis. Although the exact mechanisms underlying diabetic polyneuropathy remain complex and not fully elucidated, emerging evidence from studies points towards the involvement of genetic factors in the susceptibility to this condition. These genetic studies have provided valuable insights into the potential genetic variants and pathways that may influence an individual’s risk of developing diabetic neuropathy. Numerous studies have faced limitations, such as the examination of specific genes in small cohorts and predominantly working within Caucasian populations. Additionally, the inclusion criteria were restricted to studies published in English. Moreover, notable heterogeneity was noted among certain meta-analyses. Finally, mild publication biases were identified in particular instances. For example, several studies have looked into the relationship between the *MTHFR* gene polymorphism and the risk of diabetic neuropathy, yielding varying and inconclusive findings. Moreover, due to the limited number of studies and insufficient sample sizes examining the polymorphisms of the *GSTT* gene, confirming the association between diabetic neuropathy and either of the polymorphisms is challenging. Nevertheless, despite the progress made in understanding the genetic basis of diabetic polyneuropathy, the available data are still limited, and further extensive investigations, including whole-exome and genome-level studies, are required to validate and expand upon these findings. At this time, rs604349 in MYBPHL and rs2032930/rs2032931 in the *RMI2* gene appear to be of importance in increasing the risk for developing neuropathy, while rs917778 in MVB12B and rs2234753 in the *RXRA* gene might reduce the likelihood of this complication. The role of transketolase genetic variability could also be of significance. The utilization of these genetic variants might be useful in future genetic testing. By conducting more in-depth genetic analyses, researchers aim to uncover new genetic variants associated with DPN that could clarify its elemental pathomechanisms. In the future, the identification of specific genetic markers associated with diabetic polyneuropathy holds great promise for improving diagnostic accuracy, risk prediction, and the development of targeted therapeutic interventions. By unraveling the genetic background of diabetic polyneuropathy, researchers aim to not only improve our understanding of this complication but also prepare the way for personalized medical approaches that may lead to more effective treatments and management strategies for individuals affected by diabetic neuropathy. In conclusion, ongoing and future research efforts focused on investigating the genetic sensitivity to diabetic polyneuropathy are important for advancing our knowledge of this complicated complication, eventually aiming to improve patient outcomes and quality of life through precision treatment approaches.

## Figures and Tables

**Table 1:** Major genes that might influence the development of diabetic neuropathy.

Gene	Variant Type	Role	Publication
*Angiotensin-converting enzyme (ACE)*	homozygous DD genotype of the I/D polymorphism	Determines ACE activity and serum levels of ACE	[15]
*MTHFR* gene	C677T polymorphism	Elevates homocysteine levels	[16]
*GSTM1* and *GSTT1* genes	homozygous deletion (null genotype)	Reduces enzyme activity (GST protects against endogenous oxidative stress and exogenous potential toxins) and leads to cytogenetic damage	[17]
*GLO1* gene	CC genotype	Glo-11 reduces the formation of advanced glycemic end-products (AGEs)	[18]
*APOE* gene	Ɛ4 allele	Plays a role in the cholesterol and triglyceride metabolism	[19]
*TCF7L2* gene	rs7903146, rs7901695, rs12255372	Affects the lipid metabolism and glucose homeostasis	[20]
*VEGF* gene	C and T alleles	Determines the level of VEGF, which facilitates the proliferation of vascular endothelial cells	[21]
*IL-4* gene	VNTR	IL-4 is a cytokine that impacts immune cell chemotaxis and anti-inflammation	[22]
GPX1	rs1050450, C > T	Reduced antioxidant activity	[55]
*eNOS* gene	rs2070744 (786 T/C) rs1799983 (894 G/T)	Leads to endothelial dysfunction through the change in the synthesis of nitric oxide	[23]
*ADRA2B* gene	I/D polymorphism	Associated with autonomic dysfunction and increased sympathetic nervous system activity	[24]
*MIR146A*, *MIR128A* *MIR499A*	rs2910164 (G > C) rs11888095 (C > T) rs3746444 (GG genotype)	Associated with the level of mitochondrial DNA	[25]
*SLC19A2, SLC19A3* encoding THTR1 and THTR2		Intracellular transport of thiamine	[26]
*Transketolase* gene	rs7648309 rs63355988	Loss of protective action in the prevention of diabetic neuropathy	[27]
*Glo1* gene	rs1130534 rs1049346	Loss of defense against AGE formation	[28]
*Voltage-dependent Na channel beta-2 subunit of Nav1.7*	aspartic acid–aspartic acid mutation (D109N)	Hyperexcitability of posterior ganglion neurons	[29]
*ANO3* gene	mis-sense heterozygous variants	Increased pain sensitivity	[30]
*HCN1* gene	mis-sense heterozygous variant	Increased pain sensitivity	[30]
*TRPA1*	loss-of-function mutation	Increased pain sensitivity	[30]
*TRPV1* and *TRPV4* genes		Painless diabetic neuropathy	[30]
*SCN9A, SCN10A*, and *SCN11A*	gain-of- function mutations	Neuron hyperexcitability	[31]
Polymorphisms in the *GFRA2* gene	rs4872521 rs4872522 rs10098807 rs11774105 rs17428041 rs17615364 rs11776842 rs12545534 rs11780601	Role in the differentiation and survival of neurons	[56]
*ALR2* gene	106C/T polymorphism in the promoter region	Role in nerve conduction velocities	[32]
*ALR2* gene	50-(CA)n microsatellite polymorphism (Z + 2, Z − 2)	Susceptibility or defense against diabetic neuropathy	[57]
*GPx-1*	(rs1050450) 599C/T	Susceptibility to diabetic neuropathy	[33]
*CAT*	262C/T	Susceptibility to diabetic neuropathy	[33]
Chromosomal loci 1p35.1 and 8p21.3.		Neuropathic pain	[58]
Gene polymorphisms of *ACE, MTHFR, APOE, ALR2, GPx-1, NOS3, CAT,* and *VEGF*		Susceptibility to diabetic neuropathy	[59]
*GLP-1, PTEN, insulin, RAGE, HSP27, CW22,* and *DUSP1* in the phosphatidylinositol 3-kinase/phosphorylated protein kinase B [PI3/pAkt] signaling pathway		Possible therapeutic targets	[34]
*RMI2* gene *MYBPHL* gene *MVB12B* gene *RXRA* gene	rs2032930, rs2032931 rs604349 rs917778 rs2234753	Alters the risk of developing diabetic neuropathy	[35]

## Data Availability

Not applicable.
